# Explore How Online Healthcare Can Influence Willingness to Seek Offline Care

**DOI:** 10.3390/ijerph19137925

**Published:** 2022-06-28

**Authors:** Chensang Ye, Cong Cao, Jinjing Yang, Xiuyan Shao

**Affiliations:** 1 School of Management, Zhejiang University of Technology, Hangzhou 310023, China; 201906050630@zjut.edu.cn (C.Y.); 201906050629@zjut.edu.cn (J.Y.); 2 School of Economics and Management, Southeast University, Nanjing 211189, China; xiuyan_shao@seu.edu.cn

**Keywords:** online healthcare, offline medical willingness, channel integration, multidimensional model, PLS-SEM

## Abstract

With the recent development of internet healthcare, many hospitals have laid out their online platforms. However, there have been some poor service levels and low quality. The frequency of such problems has led to a decline in patient satisfaction. Therefore, it is vital to explore how hospitals can improve user satisfaction and willingness to visit them offline by setting up an online presence. Most studies conducted so far have remained limited to the single dimension of online or offline healthcare, with few studies exploring the relationship between them. While a few studies have explored the impact of online service quality on willingness to seek offline care, they also face the problem of a single perspective of analysis. Therefore, this study constructs a multidimensional model of the factors influencing online healthcare users’ willingness to seek offline care by integrating the value-based adoption model and the stimulus–organism–response model. Through a partial least squares-structural equation modelling analysis of 283 valid samples, this study found that online doctor–patient interactions and service quality positively impact user perception. This paper explores the development path of online healthcare from a new theoretical perspective. In addition, the findings provide new guidelines for hospitals to achieve economic and social benefits.

## 1. Introduction

With the increasing popularity of the Internet in recent years, all industries have been focusing on conducting corporate business operations on the Internet through convenient, fast and rich applications and platforms. The healthcare industry is also looking to the Internet to digitalize its businesses. Furthermore, healthcare services worldwide are leveraging emerging technologies to develop and integrate their online and offline businesses. With the rapidly developing economy, people’s income is increasing yearly—which, in turn, is increasing their consumption power and demand. In 2021, for example, China’s per capita disposable income kept increasing at a rate of 9.1%. Per capita, consumer spending was US $3700 (RMB ¥ 24,100), which amounts to a growth rate of 13.6%, with a significant increase in national consumption capacity. Moreover, the rising demand for healthcare and the increasing emphasis on health have provided a broad base for the development of the online healthcare industry.

The mobile internet is changing people’s lives and creating new opportunities for the informatization of healthcare services. Most current internet healthcare is based on an online–offline business model. Patients seek targeted treatment solutions through online medical consultation services and receive professional treatment at offline hospitals based on the online consultation recommendations. Alternatively, after an offline hospital visit, patients further take consultations online and provide feedback on the quality of the doctor’s service [1]. In addition, with the continuous development of internet healthcare, many hospitals have started laying out online healthcare platforms to optimize the allocation of healthcare resources. This can further improve the efficiency and quality of healthcare services. As a consequence of the Covid-19 pandemic, many patients have shifted from a single offline visit to multi-channel online and offline access. These changes have contributed to a dramatic expansion in the scale of internet healthcare consumption. Data from the Statistical Report on the Development Status of the Internet in China released by the China Internet Information Center 2021 show that, as of June 2021, the size of China’s online medical netizens was as large as 239 million. This figure showed an increase of 24.53 million from December 2020, accounting for 23.7% of overall netizens.

Although internet healthcare is growing, compared to traditional healthcare services, its business is still at an early development stage due to technical limitations. The online business layout of hospitals faces many other problems, such as the low level of online medical services and the information asymmetry between doctors and patients. These problems can damage the hospital’s reputation and lead to a decline in patient satisfaction with their offline visit. Effectively explaining the impact of online healthcare on patients’ satisfaction with offline care, as well as the underlying mechanisms, is central to how hospitals lay out their future online business development. The factors influencing users’ willingness to adopt online healthcare have been extensively discussed. For example, Cocosila and Archer [2] demonstrated that internal incentives and perceived risk positively influence the willingness to adopt online healthcare services. Yi et al. [3] confirmed, through an empirical study, that perceived information quality and perceived risk affect patients’ trust in online healthcare and, thus, their willingness to use it. In addition, the impact of integrating online and offline healthcare services on hospitals has been extensively studied. For example, as indicated by Huang, et al. [4], such integration can improve the professional reputation of hospitals.

The research so far has focused on patients’ decisions to seek care in a single online or offline setting. However, few scholars have considered the impact of users’ online healthcare use on their willingness to seek care offline, and few have studied the relationship between the quality of online and offline healthcare services. Moreover, the specific role and moderating mechanisms of the impact of online healthcare on the willingness to seek offline care have been rarely explored. Based on the current situation of internet healthcare, this study integrates the value-based adoption model (VAM) and stimulus–organism–response (SOR) model to investigate whether patients’ online healthcare experience influences their willingness to seek offline care and the underlying mechanisms, in order to more comprehensively understand the association between patients’ online and offline consultation behaviors.

Based on the VAM and SOR model, this study develops a model of how online healthcare influences willingness to seek offline care. Through partial least squares-structural equation modelling (PLS-SEM) analysis of the data collected through a questionnaire survey, this study explores the relationship between users’ willingness to seek offline care and their perceived characteristics of online healthcare. This study applies the VAM and SOR model to internet healthcare and explores the development path of internet healthcare from a new perspective. This research adds to the literature on online hospital operations and the integration of online and offline healthcare channels. Moreover, this study provides design insights for the hospital’s online platform’s operation and offers new ideas for improving the doctor–patient relationship. The study results can help improve patient satisfaction and the efficiency of offline visits. Finally, the findings provide new guidelines for hospitals to achieve economic and social benefits.

The rest of the paper is organized as follows. Section 2 reviews relevant research on online healthcare platforms and the impact of the online market on offline markets. Section 3 presents the relevant hypotheses and research models. Section 4 describes the demographic characteristics of the survey participants and the research methodology adopted. Section 5 presents the data analysis. The paper’s final section discusses the contributions and limitations of this study and future research directions.

## 2. Theoretical Background and Research Basis

### 2.1. Internet Healthcare Industry

Recently, the market for internet-based healthcare has been growing in some developed countries. In addition, there has been increasing research on, and application of, telemedicine for intelligent terminals, such as mobile phones. In 2009, researchers in the United States established a stroke treatment and care system to provide timely treatment to those suffering from acute strokes through telemedicine [5]. In 2012, Germany provided diagnostic services and remote treatment for dermatological patients by establishing a tele-dermatology information system [6]. In contrast, developing countries are lagging behind in this field. In Asia, South Korea was an early researcher in mobile health applications, with the development of the stroke net, which uses real-time audio-video technology to enable an essential remote diagnosis service for stroke patients [7]. A new mobile health system has also been studied in Brazil to improve home healthcare and patient treatment [8].

Today, internet healthcare is penetrating, impacting and changing traditional healthcare services. However, it is also facing many developmental problems. First, the quality of internet medical services varies. For example, most internet medical services have a single function [9]. In addition, some internet medical services lack the participation of healthcare professionals and hospital support in providing professional guidance to patients [10].

### 2.2. Online Healthcare Platform Usage Behaviour

With the development of the internet healthcare industry, hospitals are laying out their online platforms. The way doctors provide services to patients is changing [11,12], which is mainly reflected in the increasing number of doctors participating in online healthcare platforms to provide healthcare services and non-face-to-face interactions with patients [13,14]. The boom in online healthcare platforms has also led to extensive research on behavior and willingness to use online healthcare platforms. For example, McMullan [1] found that patients use online healthcare platforms to, on the one hand, gain knowledge of health and wellness and, on the other hand, find information to determine their condition. Moreover, online healthcare platforms can be used to rate or give feedback on the doctor’s offline consultation services. The role and value of online healthcare platforms have also been extensively researched. For example, doctors’ participation in online healthcare platforms can help patients make better healthcare decisions [13,15] and effectively alleviate the inequality gap in healthcare resources between urban and rural areas [16,17]. In addition, online healthcare can improve doctor–patient relationships [18,19]. Consequently, an increasing number of medical institutions and doctors are actively participating in the construction and services of online medical platforms to meet the medical needs of different patients [14].

### 2.3. Online to Offline

In the early stages of traditional e-commerce research, scholars tended to study online and offline markets separately, failing to realize that online platforms are a vast expansion of traditional offline physical industries [20,21]. Recently, scholars have started exploring users’ consumption behavior in multiple channels and realizing that the online and offline channels have a mutually reinforcing effect on each other [22]. Similarly, online healthcare and traditional offline healthcare models have synergistic and complementary relationships [23]. Therefore, they need to be explored in conjunction with each other. According to the literature, in expanding from offline to online platforms, users might change their perceptions regarding the same products and services [24]. Some recent studies, such as that of Lu and Wu [25], have suggested that online consultation services complement offline services, effectively improving the efficiency of healthcare services and enhancing consumer perceptions of service quality. Li, et al. [26] found that doctors’ online ratings and platform activity can positively influence the number of patients asking for advice at offline healthcare facilities. In addition, some scholars have explored the role of online healthcare services in facilitating offline healthcare. Yang, et al. [27] indicated that the information evaluated on online healthcare platforms can influence patients’ offline healthcare decisions.

The above literature review shows that consumer behavior and willingness to use online healthcare have been studied in depth and confirm the positive significance of online healthcare services for users. Owing to the recent popularity of online healthcare services and the influence of policy support, online and offline activities are gradually being combined through exploration of the interaction between online and offline healthcare. However, the research so far has neither explored the specific influences of online healthcare on willingness to seek offline care, nor has it provided insight into the mechanisms underlying the influence of online healthcare on offline healthcare. The present study models this influence by using the VAM and SOR model. By considering several factors, such as perceived benefits, individual subjectivity, emotional experience and perceived trust, we explore the impact of users’ online healthcare usage experience on their willingness to seek offline care. This study further enriches the relevant findings in online hospital operations management, particularly the research theory and literature on integrating online and offline healthcare channels. In addition, this study has practical significance for improving the efficiency of offline consultation and patient satisfaction and provides new ideas and perspectives for hospitals to achieve economic and social benefits.

## 3. Research Model and Hypothesis

Kim, et al. [28] proposed the idea of maximizing user-perceived value from the user’s perspective and a VAM based on the technology acceptance model (TAM) and perceived value theory. TAM aims to investigate the relationships among individuals’ beliefs, attitudes, intentions and actual behavior in organizational settings [29]. This model appropriately explains individuals’ receptive behavior towards systems and applications in organizational settings. However, with the development of mobile internet and m-commerce, acceptable behavior has become more responsive to consumer choice than organizational advice [28]. Consumers now focus on whether goods can satisfy their specific value needs in the voluntary consumption process. Based on this, Kim proposed a VAM to analyze users’ adoption intentions and behaviors in the mobile internet environment.

Meanwhile, the SOR model was proposed by Mehrabian and Russell [30] in 1974. The authors argued that environmental factors stimulate individuals’ psychological and behavioral responses. This theory also states that environmental factors, as a stimulus (S), first affect an individual’s psychological state (O), and these psychological changes can further influence the individual’s response behavior (R).

**Figure 1:**
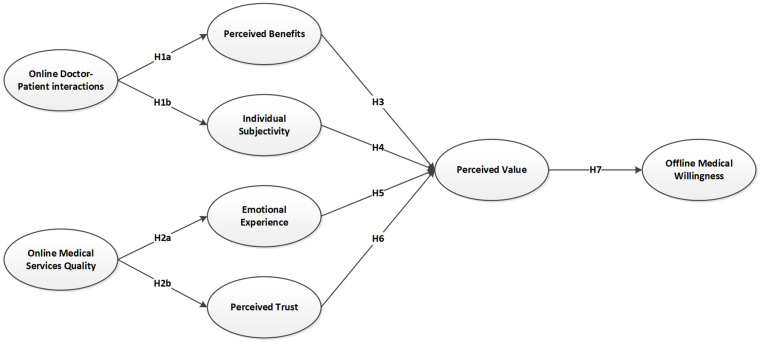
Research model.

This study integrates VAM into the SOR model framework to explore how online healthcare affects patients’ psychological and subsequent offline visit behavior. Since patients’ online consultations can impact their offline consultation experience, this study uses doctor–patient interactions and service quality in the online healthcare environment as the stimulus (S). As the stimulation of the online environment causes changes in the user’s psychological state, we use patients’ perceived benefits, individual subjectivity, emotional experience, perceived trust and perceived value as the organism (O). After the online consultation and corresponding change in the psychological state, the patients’ willingness to seek offline care is used to predict the likelihood of their subsequent responsive behavior (R). We select two elements of VAM—perceived benefits and perceived value—and integrate them into the SOR model framework. Based on this, we model how online healthcare influences willingness to seek offline care, as shown in Figure 1.

According to e-commerce research, online communication between consumers and salespeople enhances their ability to grasp information about the purchase process and to better understand the product or service [31]. For the online healthcare environment, Umar et al. [32] revealed, through an empirical study, that collaborative doctor–patient interaction behavior positively affects medical diagnostic services. In addition, many studies have confirmed that online healthcare interactions can increase patient initiative and engagement. For example, Dunn [33] demonstrated that online healthcare effectively reduces patients’ perceived interpersonal risk and increases their willingness to self-disclose complex issues. May [34] pointed out that online doctor–patient interactions increase patients’ sense of control and involvement in treatment planning. The above studies have shown that effective doctor–patient interactions can reduce information asymmetry between doctors and patients and can help patients better understand their health status and make more rational treatment decisions [35,36]. Based on this, we propose the following hypotheses:

**H1a.**  *Online doctor–patient interactions have a significant positive effect on perceived benefits*.

**H1b.**  *Online doctor–patient interactions have a significant positive effect on individual subjectivity*.

Following Zeithaml et al. [37], web service quality is defined as the efficiency and effectiveness of product or service delivery. Service quality comprises content process quality and outcome quality [38]. Content process quality refers to consumers’ overall evaluation of a website’s content and performance, including website design, security and privacy. Service outcome quality refers to consumers’ evaluation of whether the online service has met their expectations. When applying the above service quality classification to the present study, service quality includes, on the one hand, a patient’s evaluation of the design and safety of the online platform and, on the other hand, evaluation of the experience level of the online doctor and whether the information provided meets the patient’s expectations. Many recently conducted empirical studies have shown that the quality of online services has a significant and direct positive impact on customer trust [39,40,41]. Many e-commerce studies have confirmed that a web design’s professionalism, convenience and security can enhance consumer satisfaction and trust and yield positive sensory emotions and a pleasant shopping experience [42]. Hence, it is hypothesized that

**H2a.**  *Online medical service quality has a significant positive impact on emotional experience.*

**H2b.**  *Online medical service quality has a significant positive impact on perceived trust*.

According to the definition of perceived value [43], consumers compare the expected benefits and costs of a product or service and, thus, perceive a value for this transaction. The expected benefits of a product or service are an essential part of the perceived value. Some researchers have studied usage behavior in the mobile internet context from the perspective of perceived value maximization [28]. The results indicated that the magnitude of perceived value depends on both perceived benefits and losses. Perceived benefits include the usefulness of a product or service and its emotional value. In this study, the user perceived benefits are mainly derived from the helpful information provided by online healthcare and emotional relief for an individual. Thus, we speculate that if online healthcare meets the expected benefits for users, it can increase their perceived value. Thus, we hypothesized that

**H3.**  *Perceived benefits have a significant positive effect on perceived value*.

The traditional doctor–patient relationship is paternalistic, with the doctor dominating the consultation process [44]. With the development of online healthcare, the communication between doctors and patients gradually shows a consumer [45] and consultative mode [46]. In this mode, patient involvement and the information available are increased [47]. Studies have shown that, if the patient is dominant during the consultation, their perceived benefit is also enhanced [34]. According to the previous hypothesis, perceived benefits further influence perceived value. On this basis, we propose the following hypothesis:

**H4.**  *Individual subjectivity has a significant positive effect on perceived value*.

The relationship between emotional experience and consumer-perceived value in e-commerce has been extensively studied. For example, in a mobile instant messaging service study, perceived value was classified into four dimensions: functional, emotional, social and monetary values [48]. Emotional value refers to the emotions of pleasure and delight experienced when consumers use a service. In a study of website efficacy and entertainment experiences, emotional factors were found to be more influential than information technology factors when consumers adopted online shopping [49]. According to Keeney [50], emotional experiences are a vital dimension influencing online shopping attitudes when comparing consumers’ experiences before and after online shopping. These findings indicate that online doctor–patient interactions and patient-centered care provide a positive emotional experience for users, influencing their perceived value. Hence, we hypothesized that

**H5.**  *Emotional experience has a significant positive effect on perceived value*.

Jarvenpaa et al. [51] argued that when a consumer’s trust in an online merchant increases, their perceived risk decreases, while their perceived benefit increases. Jones and Leonard [52] classified the factors affecting consumer trust in an e-commerce environment as website quality, information asymmetry and online security. They argued that the privacy and security of online healthcare platforms, the level of service provided by online doctors and the reliability of the information provided considerably influence user trust, affecting user benefits and value perceptions. Thus, we propose the following hypothesis:

**H6.**  *Perceived trust has a significant positive effect on perceived value*.

Choi et al. [53] confirmed the significant effect of customers’ perceived value on their satisfaction and behavioral intentions. Due to the information asymmetry between doctors and patients, patients tend to prefer hospitals or doctors with widely recognized healthcare service quality when making offline healthcare decisions [54]. In addition, the information obtained from online healthcare platforms can provide a basis for offline patient access and improve the efficiency of patient access [55]. When patients are at an information disadvantage, they tend to choose offline hospitals based on their satisfaction or the perceived value of online visits. Therefore, it is hypothesized that a positive experience and valuable information gained by patients on an online healthcare platform will lead them to prefer this hospital for offline consultations. Meanwhile, the halo effect can also explain the correlation between online and offline behaviors. It is a factor that influences interpersonal perceptions and refers to people’s subjective impressions when making biased generalizations [56]. Thus, we hypothesize that

**H7.**  *Perceived value has a significant positive effect on offline medical willingness*.

## 4. Methodology

### 4.1. Sample

This study explores how online healthcare can improve user satisfaction and willingness to seek offline care. Therefore, we focus on users who have used online healthcare platforms to understand their attitudes towards their use of online healthcare platforms and their subsequent usage behavior. Our data collection dates are from September 2021 to December 2021. In general, this period is not the pre-epidemic phase of the outbreak, and the outbreak situation in many countries is stabilizing. This has somewhat weakened the shift in user attitudes and behavior towards online healthcare due to the epidemic. First, we constructed the model and initial questionnaire for this study based on VOM and SOR theories and existing research findings. To validate the proposed model, we collected empirical research data on the satisfaction of users’ online healthcare experience on offline visits using an online questionnaire distributed to users of online healthcare platforms. The platforms chosen for this study are the three mainstream online medical platforms in China, namely Good Doctor Online, Ping An Good Doctor and Ding Xiang Doctor. The main differences between these three platforms lie in their branding and interface design. However, they are similar in service functions, mainly including online booking, online consultation, doctor-patient interaction and health management. Take the doctor-patient interaction function as an example. All three platforms can achieve one-to-one online interaction between doctors and patients and regularly carry out activities such as live streaming of famous doctors. We invited users who had used Good Doctor Online, Ping An Good Doctor or Ding Xiang Doctor to respond to the questionnaire by sending them an email. The invited users only needed to have used any of these three platforms. We also surveyed by distributing electronic questionnaires in health care facilities and communities. Before formally collecting the data, we conducted a pre-survey by randomly selecting 30 people from the online platform to validate the questionnaire. The questionnaire’s composition was revised through discussions with some academics and experts. We also set up a pre-censored mechanism for the questionnaire. The respondents were required to have at least one account with an online provider and to have had at least one online consultation or one offline visit in the last six months. To validate the responses, we set three questions at the beginning, middle and end of the questionnaire (i.e., bogus items), which set out the information that was clearly wrong or not valid. If selected incorrectly, this would indicate that the participant did not read the question carefully. Therefore, an invalid questionnaire would result in an incorrect answer to any of the three questions. We also excluded questionnaires that took less than 5 min to be completed, due to quality concerns.

The questionnaire for this study consists of three parts, the first of which focuses on disclosing information about this study to the respondents. We disclosed the purpose of this study to the respondents in advance, followed by the principle of voluntary participation by the respondents. In addition, we recorded information in an anonymous form and followed a strict data protection and storage policy. Only persons related to the research and authorized by a supervisor had access to the information. In addition, the subjects and researchers had no conflicting interests. Moreover, the respondents could withdraw from the survey at any time during the completion of the questionnaire. The second part of the questionnaire collected basic information about the respondents, mainly gender, age, and the online healthcare platform visited. The third part of the questionnaire was a survey of questions related to this study, and we designed 22 question items for eight variables. Please see Appendix A for the questionnaire items, definitions and sources.

We collected 361 questionnaires in total. After eliminating invalid questionnaires (through the pre-screening mechanism, fraudulent selection of questions and response time), 283 valid questionnaires were obtained. We developed a final questionnaire in accordance with the existing literature. The study sample comprised 39.22% females and 60.78% males, with the leading age group concentrated between 35 and 54 years (50.18%). Online medical users are mainly middle-aged people, whose health is more unstable and who are more concerned about health and wellness. Therefore, the returned questionnaires broadly corresponded to the current profiles of the main user groups. The complete characteristics of the respondents are presented in Table 1.

### 4.2. Measures

All relevant variables measured in this study were taken from previous studies and adjusted for the context of online and offline healthcare. We evaluated eight variables: online doctor–patient interaction, online medical service quality, perceived benefits, individual subjectivity, emotional experience, perceived trust, perceived value and willingness to seek offline care. The questionnaire comprised a Likert scale ranging from 1 (strongly disagree) to 7 (strongly agree); the respondents were asked to select a number from the scale.

To evaluate online doctor–patient interactions, we drew on a scale designed in [57], mainly through user ratings and forum discussions. Two scales were then used to measure perceived benefits [51,58], which comprised two main components: the fact that the information obtained from online healthcare was in line with the patients’ expectations and that the online doctor was trustworthy. Perceived trust was measured by referring to the scale designed in [51], depending on whether the online doctor’s services met the patient expectations and whether the online healthcare platform prioritized the patient’s interests. The online service quality scale was adapted from [59], a scale based on the quality of information system services. Perceived value was adapted from the scale designed in [43]. In addition, individual subjectivity was measured using three items [47] related to the patient’s communication strategy with the doctor, the patient’s list of questions and the frequency at which the patient asks the doctor to explain the treatment procedure in detail. For emotional experience, the scale proposed in [48] was referenced and adapted. Willingness to seek offline care was measured using a scale designed in [60], which assesses patients’ willingness through two main items: ‘I am likely to visit a doctor in the future’ and ‘I have the desire to visit a doctor in the future’.

### 4.3. Data Analysis

We used the PLS-SEM approach, which deals with the relationships between variables [61]. In addition, the SmartPLS 3.3.7 software was used to validate the structural equation model. The proposed method can analyze complex problems with different factors and deal with variables that are not directly measurable and some that are more subjective [62]. The impact of the online healthcare experience on offline visits explored in this study involves numerous variables that are difficult to be measured accurately and directly. Therefore, directly measurable exogenous indicators are required to represent these variables. SEM methods can be divided into PLS and linear structural relationship methods. The advantage of the PLS-SEM model is that it can leverage the information obtained from the data to minimize the error term. Moreover, the model does not require a large sample size, model identification problem or distribution state of the data. It can effectively deal with the covariance problem between variables. Therefore, the PLS-SEM model was used in this study for data analysis [62]. A previous study also showed that PLS-SEM is better for assessing reflective and formative structures [63]. PLS-SEM combines factor analysis with path analysis and can analyze multiple influencing factors simultaneously, allowing for the simultaneous treatment of latent variables and their indicators [64].

**Figure 2:**
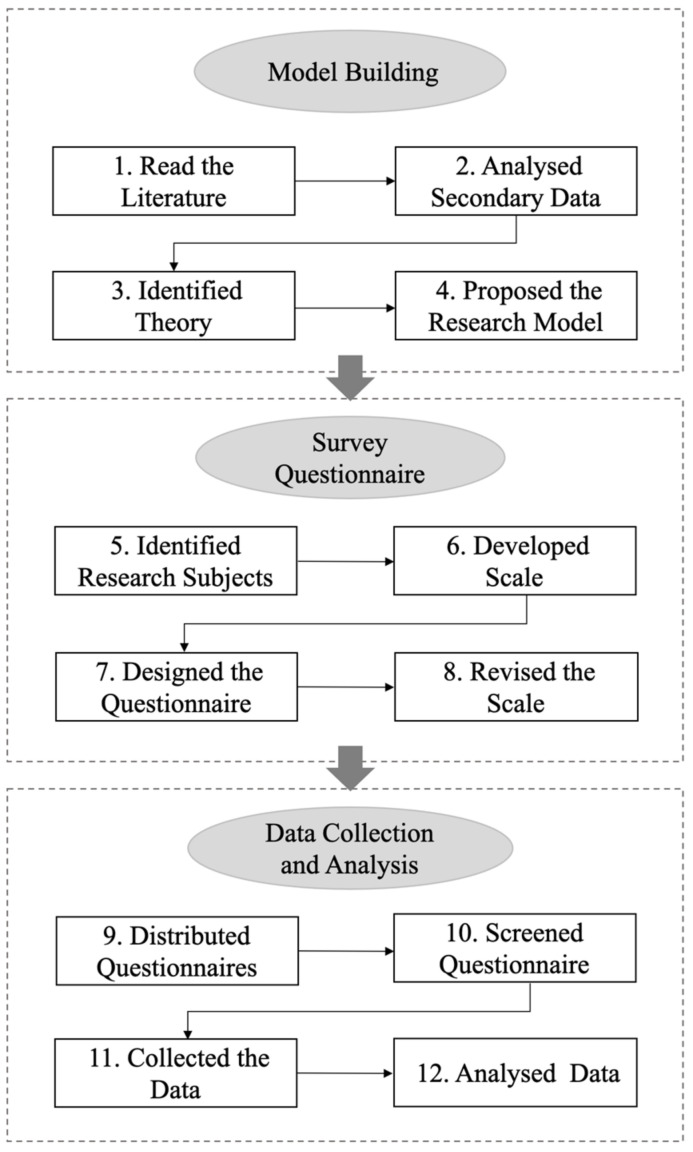
Research procedure.

In this study, data analysis was conducted in three main steps. First, a reliability test was conducted to examine internal consistency reliability using composite reliability (CR) and Cronbach’s alpha (CA). Indicator reliability was tested using factor loadings. Next, validity analyses were conducted to assess the convergent validity of the measurement model using Average Variance Extracted (AVE) and the discriminant validity of the model using the Fornell–Larcker metric and cross-loadings. Finally, the amount of explained variance in the correlation structure was assessed using the coefficient of determination (*R*^2^). In addition, we performed a path coefficient analysis and a research hypothesis test. A flow chart of the research process is shown in Figure 2.

## 5. Results

### 5.1. Measurement Model

The measurement model shows the relationship between structural and indicator variables [65]. This study assessed the measurement model in four dimensions: indicator reliability, internal consistency reliability, convergent validity and discriminant validity. We used CR and CA to test for internal consistency reliability and factor loadings to test for indicator reliability. In addition, we used AVE to assess the convergent validity of the measurement model and the Fornell–Larcker metric and cross-loadings to assess the differential validity of the model. In terms of reliability tests, as shown in Table 2, the CA values for all latent variables were greater than 0.840, and the CR values were greater than 0.900. As shown in Table 3, all measurement indicators had factor loadings greater than 0.853 on the constructs to which they belonged, indicating that our measurement model exhibited good internal consistency reliability and indicator reliability [66].

AVE is used to ensure convergent validity for the validity analysis and is expected to exceed the 0.5 level. In our measurement model, all AVEs were above 0.750, meaning that all potential constructs could explain most of the variance in their indicators, which indicates that the model has good convergent validity [66]. In addition, as shown in Table 4, the square root of AVE was greater than the correlation coefficient of the latent variable, which is consistent with the standard Fornell–Larcker hypothesis and meets the Fornell–Larcker criterion hypothesis. Thus, the model passed the differential validity test [67]. As shown in Table 3, each square root of the AVE of a latent variable was greater than the squared correlation of that variable with all other latent variables, verifying that the model passed the differential validity test [67].

### 5.2. Structural Model

**Figure 3:**
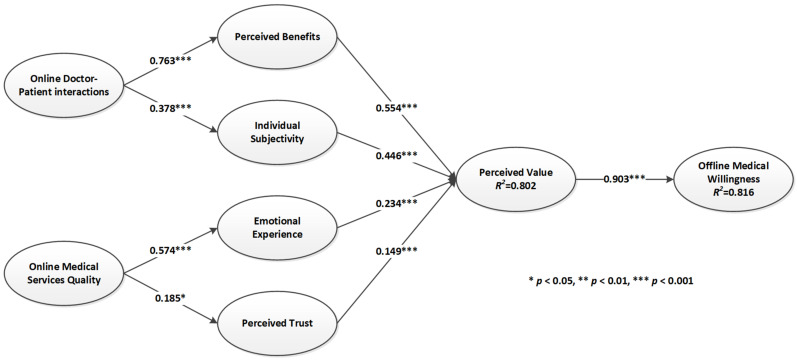
The path coefficient of research model.

The main criterion used to assess the amount of explained variance in the correlation structure is the coefficient of determination (*R*^2^). The closer *R*^2^ is to 1, the more valuable the model is in explaining the hypothesis. The model has significant explanatory power when *R*^2^ > 0.67 [68]. In this study, the willingness to seek offline care reached a high level of explanation of the model (i.e., 0.816), indicating that the proposed model exhibited good explanatory power. In addition, the *t*-value significance test of the path coefficients was performed by bootstrapping in SmartPLS with an original sample size of 283. Using a bootstrapping procedure with 5000 resamples, the path significance of all hypothesized variables was computed. The path coefficients and significance results are shown in Figure 3.

This study assessed the overall model prediction performance (both measurement and structural models) using the goodness of fit (GoF) index. The GoF value was obtained according to the formula shown in [69] and the criterion for assessing the size of the GoF effect introduced by [70]. We calculated the GoF as 0.891 based on the formula GoF = √(AVE × *R*^2^), which sufficiently validates the global model.

In addition, the path coefficients and their associated t-statistics were examined to assess the importance and relevance of the structural relationships between potential building blocks in the model. The following results were obtained (Table 5): the perceived value had a significant positive effect on willingness to seek offline care (*t* = 91.744, *p* < 0.001), perceived benefits, individual subjectivity, emotional experience and perceived trust on perceived value (*t* = 14.503, *p* < 0.001; *t* = 11.186, *p* < 0.001; *t* = 7.729, *p* < 0.001; *t* = 4.413, *p* < 0.001). Online doctor–patient interactions had a significant effect on perceived benefits (*t* = 41.007, *p* < 0.001) and individual subjectivity (*t* = 6.754, *p* < 0.001). Online medical service quality had a significant effect on emotional experience (*t* = 12.242, *p* < 0.001) and perceived trust (*t* = 2.375, *p* < 0.001). Therefore, all nine hypotheses proposed in this study passed the significance test.

### 5.3. Assessing Global Model Fit

The first step in the PLS-SEM analysis is to evaluate the global model fit [71]. When there is no fit between the data and the model, the data include additional information that the model lacks. Therefore, we conducted standardized root mean square residuals (SRMR), unweighted least squares difference and difference in geodesic tests to perform a set of bootstrap-based model fit checks [72]. SRMR is used as the estimated model fit statistic to verify whether there is a significant disagreement between the conceptual model and the empirical correlation matrix [73]. In this case, the critical level for achieving a satisfactory model fit in PLS-SEM is 0.08 [71]. In our model, the SRMR reached an acceptable value of 0.035.

## 6. Discussion

We constructed a model based on the SOR and VAM theories to determine the influence of online healthcare on willingness to seek offline care. In addition, we conducted an empirical study by distributing questionnaires online. The following findings and test results were obtained.

First, the results of the data analysis indicated that online doctor–patient interactions have a significant positive effect on perceived benefits and individual subjectivity. This is in line with the findings of several researchers on doctor-patient interactions [34]. Good doctor–patient interactions involve both information disclosure by the patient to the doctor and information support given by the doctor to the patient. On the one hand, online doctor–patient interactions enable doctors to provide more accurate advice and create value for patients. On the other hand, positive doctor–patient interactions not only enable patients to lower their defenses and increase their sense of security but also increase patient engagement throughout the healthcare process and ease tensions in the doctor–patient relationship [74]. Users are given ample time to communicate and give feedback when interacting with their doctors online. In the process, patients are better informed about their condition, and therefore the perceived benefits are enhanced accordingly. The online, non-face-to-face form of communication also increases patients’ sense of security, allowing patients to be more forthcoming in disclosing information involving personal privacy, which often becomes crucial to the doctor’s diagnosis [32].

Second, online healthcare service quality significantly affects emotional experience and trust. In this paper, online healthcare service quality is defined as the design features of the online platform, security and privacy and the level of competence of online doctors. As mostly middle-aged and older people use online healthcare platforms, the ease of use and simplicity of the interface design of the platform is crucial. It is the primary requirement for patients to be able to use the online healthcare platform conveniently. At the same time, due to the unique nature of healthcare platforms, patients’ privacy and account security are often involved, so it is also crucial that the platform’s privacy and security mechanisms are in place. The findings show that the interface design and privacy protection of online healthcare platforms can make users feel safe and secure when using online healthcare platforms. It increases users’ trust in the platform and provides a pleasant emotional experience. This finding also proves that an aesthetic design of an e-commerce website is essential for improving patients’ experience during online medical consultations [75]. In addition, online doctors’ qualifications and service level are other essential dimensions of service quality. Doctors with relevant titles and good service attitudes are more likely to gain patients’ trust [39].

Third, perceived benefits, individual subjectivity, emotional experience and perceived trust significantly affect the perceived value. On one hand, users find it valuable to engage in online healthcare if they perceive that the services provided by online healthcare can yield helpful information and provide relief to their emotions. This finding further supports the research findings on users’ usage behavior in the mobile Internet context, namely that the magnitude of users’ perceived value depends mainly on perceived benefits. The perceived benefits mainly include the usefulness value of using a product or service and the pleasure of this usage behavior on an emotional level [28]. On the other hand, the patient’s initiative and willingness to self-disclose are increased using the online doctor–patient interaction model. During this process, patients can ask the doctor about their treatment and gain a more comprehensive understanding of their condition. Consequently, their satisfaction and perceived value are increased accordingly. This also reaffirms that doctor–patient interactions can facilitate the effectiveness of medical diagnostic services [32]. In addition, when online medical advice provides positive emotional support to patients, it can improve their perceived value. While medical consultations were previously perceived as a cold, passive process, the findings of this paper confirm that patient satisfaction is greatly enhanced when patients are given some emotional comfort and support during the consultation process. Further evidence is that the impact of emotional experience on online purchase intentions in e-commerce is also applicable to the online healthcare sector [49]. In addition, as argued in the previous section, the privacy and security of online healthcare platforms, the level of service provided by online doctors and the reliability of the information provided significantly influence user trust. The study findings confirm that user trust can significantly influence patients’ perceived value. This is yet another indirect illustration of how the security of online medical platforms and the level of service provided by online doctors can influence patients’ perception of value.

Fourth, perceived value has a significant positive impact on willingness to seek offline care. In the traditional offline healthcare model, patients are always at a disadvantage in terms of information. Online healthcare helps change the information asymmetry between doctors and patients. Therefore, patients can use their experience of online healthcare to assess the quality of the doctor’s service or the professionalism of the hospital as a basis for choosing a hospital or doctor to visit offline [27]. On the one hand, patients can use the triage function of online healthcare to position themselves for offline medical treatment, thereby improving the efficiency of offline medical treatment. On the other hand, the level of service provided by the doctors on the online medical platform represents the image and standard of the hospital they work in. If patients have an excellent online experience, they will be encouraged to seek further treatment offline. Suppose patients do not perceive the value and benefits of online healthcare. In that case, they will also perceive the quality of the doctor’s hospital as mediocre under the influence of the halo effect [55].

Compared to existing papers, this paper makes theoretical contributions in the following areas. First, the available research provides insight into the issue of healthcare access decisions in a single setting, either online or offline. However, it lacks in exploration of the impact of online healthcare on offline access. Therefore, this study considers multiple factors, such as perceived benefits, individual subjectivity, emotional experience and perceived trust, through a combination of factors. We construct a model of how online healthcare influences willingness to seek offline care and explored the underlying mechanisms through which the online healthcare experience influences willingness to seek offline care. Second, this study integrates VAM into the SOR model framework and explores the development path of online healthcare from a new theoretical perspective. The results add to the theory and literature related to online hospital operations and the integration of online and offline channels.

This study also has practical implications for integrating online and offline channels in the healthcare sector. First, this study empirically explores the influence of online healthcare on the willingness to seek offline care, which provides design insights for hospitals to layout the operation of online healthcare platforms. Second, this study provides new ideas for improving the doctor–patient relationship. On one hand, hospitals can enhance patients’ trust by building a high-quality and efficient online medical service platform. On the other hand, the online platform built by hospitals or in cooperation with others needs to improve the quality of doctors’ services, enhance patients’ subjectivity and participation and bring patients an excellent emotional experience and perception of benefits. In turn, this will increase the willingness of patients to visit the hospital offline. Furthermore, as the current trend of homogenization of online healthcare services in China has led to a bottleneck in the growth of services, this study helps online healthcare platforms to identify consumers’ intentions better and understand users’ needs for the quality of online healthcare services and doctor-patient interactions, to improve users’ perception of value and thus their willingness and satisfaction to seek care offline. Finally, the findings of this paper provide new ideas for hospitals to realize economic and social benefits. If hospitals can develop efficient online healthcare platforms or partner with online healthcare platforms, they can bring more economic benefits to their offline business, alleviate regional healthcare imbalances and improve patient satisfaction and well-being.

Based on the above-mentioned findings, we make the following recommendations for hospitals. First, hospitals should encourage doctors to leverage the online channels to provide online consultations. They should also create incentives to improve the service quality and communication skills of doctors who consult online to meet people’s medical needs and access to care. Second, online and offline healthcare services must be integrated. Through initial diagnosis and communication online, we can understand a patient’s situation in order to improve efficiency during offline visits and reduce the queuing time. Finally, the online consultation platform is optimized for ease of use. The results of our questionnaire also show that middle-aged and older people are the leading groups seeking medical treatment online. Therefore, the online consultation platform should have simple functional settings to make them easy to use. It is also essential to improve the web pages’ interface design and privacy protection to increase users’ trust and willingness to disclose their personal information. For example, the font design of the website can be made slightly more prominent and the colors can be made more subdued to suit the habits of middle-aged and older adults. In addition, a unique interface can be designed for special groups, such as the color-impaired and color-blind.

## 7. Limitations and Future Research

Although the proposed model passed the statistical test, there are still some limitations to its results. First, the online healthcare factors influencing the willingness to seek offline care involve a broader range of dimensions. The results obtained in other regions and hospitals might not be the same as those obtained in this study, due to differences in the level of care and patient needs. We did not conduct a comparative study across different markets and cultural environments. Second, the scope of medical treatment is extensive, and different medical items can affect the study results. This study did not break down the various medical items, and instead classified them under the broad umbrella of medical care. In addition, there are differences in the acceptance and perception of online healthcare among different markets. They are influenced by multiple games and interactions among hospitals, regulators, patients and their families and social media. Finally, as we are currently in the midst of a new epidemic, the epidemic may impact users’ online healthcare habits and behaviors. We did not fully consider the psychological shift in users’ attitudes towards online healthcare during the epidemic period [76,77].

Therefore, future research can be refined and deepened from the following four viewpoints. First, subsequent research could explore the moderating effect of culture, level of care and technological environment on the willingness to seek offline care in different regions and market environments. Second, we can extend the study to different healthcare programs and compare the differences in the factors influencing patients’ online access to offline visits between these programs. In addition, we can consider the interactions among hospitals, regulators, patients and social media to explore the willingness and satisfaction related to online healthcare use. Finally, we will continue to explore users’ changing attitudes and behaviors towards online healthcare in the post-epidemic era.

## 8. Conclusions

Recent research has explored patients’ healthcare needs and behaviors in a single online or offline setting, affirming the positive significance of online healthcare services for users. However, there is a relative paucity of research on the impact of online healthcare usage experiences on willingness to seek care offline. This study, therefore, uses the VAM and SOR model to construct a model of how online healthcare influences willingness to seek offline care and explores the underlying mechanism in depth from multiple perspectives. We processed and analyzed the data from 283 valid samples using PLS-SEM through a questionnaire method. The study results indicated that online doctor–patient interactions have a significant positive effect on perceived benefits and individual subjectivity. Second, the quality of online healthcare services has a significant positive effect on the effective experience and perceived trust. Again, perceived benefits, individual subjectivity, emotional experience and perceived trust significantly affect perceived value. Finally, perceived value has a significant positive effect on willingness to seek offline care.

On the one hand, this study integrates the VAM and SOR model to explore the intrinsic relationship between online healthcare experience and the willingness to seek offline care from a new theoretical perspective. Its findings enrich the literature on integrating online and offline healthcare channels. On the other hand, the findings help hospitals guide patients to offline care by laying out a high-quality online healthcare platform. Initial triage and diagnosis online can help improve the efficiency of offline patient access and enhance patients’ medical satisfaction. In addition, the hospital’s layout of an efficient online platform can alleviate the scarcity and imbalance of regional healthcare resources. The findings of this study validate that online doctor–patient interactions and service quality are critical factors for further offline visits by patients and, therefore, provide hospitals with insights for achieving both economic and social benefits.

## Figures and Tables

**Table 1:** Demographic profile of respondents (N = 283).

Measure	Category	N	Percent
Gender	Male	172	60.78%
	Female	111	39.22%
Age	<18	0	0.00%
	18–24	27	9.54%
	25–34	76	26.86%
	35–44	81	28.62%
	45–54	61	21.55%
	55–64	29	10.25%
	Over 65	9	3.18%
Education	High School	46	16.25%
	College	79	27.92%
	Undergraduate	101	35.69%
	Postgraduate	57	20.14%

**Table 2:** Descriptive statistics for the constructs.

	CA	CR	AVE
Offline Medical Willingness (Will)	0.921	0.962	0.927
Perceived Value (Valu)	0.912	0.958	0.919
Perceived Benefits (Bene)	0.902	0.939	0.837
Individual subjectivity (Subj)	0.970	0.980	0.943
Emotional Experience (Expe)	0.922	0.951	0.866
Perceived Trust (Trus)	0.916	0.947	0.856
Online Doctor-Patient Interactions (Inte)	0.841	0.904	0.759
Online Medical Service Quality (Qual)	0.957	0.972	0.921

**Table 3:** Factor loadings and cross loadings.

	Will	Valu	Bene	Subj	Expe	Trus	Inte	Qual
Will.1	**0.964**	0.883	0.645	0.643	0.305	0.459	0.801	0.459
Will.2	**0.962**	0.856	0.632	0.602	0.313	0.465	0.772	0.463
Valu.1	0.891	**0.961**	0.691	0.561	0.328	0.561	0.804	0.461
Valu.2	0.840	**0.956**	0.683	0.506	0.260	0.521	0.755	0.414
Bene.1	0.655	0.685	**0.924**	0.167	0.060	0.610	0.724	0.003
Bene.2	0.603	0.670	**0.910**	0.148	0.024	0.562	0.697	−0.044
Bene.3	0.559	0.608	**0.910**	0.049	0.039	0.515	0.672	−0.061
Subj.1	0.663	0.578	0.154	**0.971**	0.082	0.128	0.395	0.631
Subj.2	0.617	0.534	0.134	**0.975**	0.052	0.118	0.368	0.607
Subj.3	0.601	0.506	0.101	**0.966**	0.097	0.102	0.335	0.603
Expe.1	0.306	0.291	0.050	0.081	**0.936**	0.064	0.301	0.528
Expe.2	0.329	0.316	0.092	0.095	**0.930**	0.127	0.315	0.526
Expe.3	0.259	0.250	−0.018	0.045	**0.926**	0.057	0.271	0.547
Trus.1	0.453	0.520	0.569	0.115	0.066	**0.929**	0.462	0.166
Trus.2	0.459	0.550	0.564	0.129	0.105	**0.934**	0.456	0.203
Trus.3	0.419	0.495	0.580	0.086	0.075	**0.913**	0.437	0.141
Inte.1	0.738	0.724	0.680	0.369	0.266	0.454	**0.871**	0.228
Inte.2	0.719	0.730	0.674	0.317	0.303	0.398	**0.888**	0.263
Inte.3	0.675	0.671	0.639	0.300	0.262	0.423	**0.853**	0.254
Qual.1	0.454	0.437	−0.044	0.623	0.549	0.192	0.265	**0.964**
Qual.2	0.437	0.414	−0.054	0.594	0.547	0.150	0.252	**0.953**
Qual.3	0.486	0.464	−0.007	0.604	0.556	0.191	0.301	**0.962**

Note: Bold numbers indicate outer loading on the assigned constructs.

**Table 4:** Correlations among constructs and the square root of the AVE.

	Will	Valu	Bene	Subj	Expe	Trus	Inte	Qual
Will	**0.963**							
Valu	0.903	**0.959**						
Bene	0.663	0.717	**0.915**					
Subj	0.647	0.557	0.135	**0.971**				
Expe	0.321	0.307	0.045	0.079	**0.930**			
Trus	0.480	0.565	0.616	0.120	0.089	**0.925**		
Inte	0.817	0.814	0.763	0.378	0.318	0.488	**0.871**	
Qual	0.479	0.457	−0.036	0.633	0.574	0.185	0.284	**0.959**

Note: Bold numbers represent the square roots of the AVEs.

**Table 5:** Path Coefficients.

	Original Sample (O)	Sample Mean (M)	T Statistics (|O/STDEV|)	*p* Values
Valu -> Will	0.903	0.904	91.744	0.000
Bene -> Valu	0.554	0.553	14.503	0.000
Subj-> Valu	0.446	0.444	11.186	0.000
Expe -> Valu	0.234	0.234	7.729	0.000
Trus -> Valu	0.149	0.151	4.413	0.000
Inte -> Bene	0.763	0.766	41.007	0.000
Inte -> Subj	0.378	0.381	6.754	0.000
Qual -> Expe	0.574	0.573	12.242	0.000
Qual -> Trus	0.185	0.192	2.375	0.018

## Data Availability

The data presented in this study are available on request from the corresponding author. The data are not publicly available due to the privacy restrictions.

## Appendix A. Ethical Review

The empirical study was conducted to investigate the participants’ intentions for specific behaviors; thus, ethics is an essential consideration of this study. To ensure the rights of the participants, this study took the following steps:

First, the research was conducted under the guidance of two supervisors with extensive research experience, particularly Assoc. Prof. Cong Cao, Ph.D., who has years of practical experience in empirical research. Thus, the study was designed and conducted in a relatively reliable environment.

Second, the researcher had a detailed discussion with the School of Management, Zhejiang University of Technology. Based on their advice, improvements were made to the forms, information letters, and letters of consent.

Third, after completing the design of the empirical study, the researcher submitted a research proposal to the School of Management, Zhejiang University of Technology, as required, expounding upon the research arrangements, explaining how to guarantee the rights of the study participants, and other matters related to the process of data collection.

Fourth, the researcher strictly followed relevant institutional requirements when conducting the empirical study. For example, the researcher informed the participants in detail about the research before it began, including, but not limited to, the research purpose, methods and process. The researcher also stressed the unpaid and voluntary nature of participating in the research study, so the participants would have the right to refuse to participate or withdraw from participating at any time. The researcher only began data collection after obtaining the consent of the participants.

Fifth, all data were collected anonymously and stored on a designated computer. The data could not be copied and/or distributed at will, and the computer was also controlled by a password to ensure that only the researchers involved in the experiments had access. The data were encrypted on a hard disk to ensure against the theft or loss of the computer, thus protecting the data and the privacy of the participants, to a large extent.

## Appendix B. Measurement Items

**Table A1:** Questionnaire Items.

Factors	Definition	No. of Items	Items	Source
Offline Medical Willingness	The willingness and behavior of patients to make offline hospital and doctor choices following information obtained from online healthcare platforms.	2	1. I am likely to visit a doctor in hospital in the future. 2. I have the desire to visit a doctor in hospital in the future.	[60]
Perceived Value	The individual’ s overall evaluation of perceived benefits and cost effectiveness in obtaining products or services.	2	1. Online healthcare platforms can make my life easier. 2. Online healthcare platforms can solve my disease consultation problems.	[43]
Perceived Benefits	The sum of the material and spiritual benefits felt by the customer in the transaction or through consumption.	3	1. The information obtained from online healthcare was in line with my expectations. 2. The online doctor was trustworthy. 3. Using the internet to consult on this platform is a good idea.	[51,58]
Individual Subjectivity	Active engagement behavior of patients during online consultations	3	1. I will proactively ask questions to doctors on healthcare platforms. 2. I will ask the doctor to explain the procedure to me in detail. 3. I take the lead in communication with the doctor.	[47]
Emotional Experience	The emotions of pleasure and delight experienced when consumers use a service.	3	1. I feel good when I use online healthcare platform. 2. Online healthcare platform gives me pleasure. 3. Using online healthcare platform is enjoyable.	[48]
Perceived Trust	Users’ trust in the security of the online platform and in the reliability of the services provided by online doctors.	3	1. This online healthcare platform is trustworthy. 2. The online doctor’s services met my expectations. 3. I trust this online healthcare platform keeps my best interests in mind.	[51]
Online Doctor-Patient Interaction	Online communication between doctors and patients, including proactive disclosure and feedback of information.	3	1. I will disclose private information about my condition to my doctor. 2. The doctor can provide me with valuable information. 3. My communication with the doctor was smooth.	[57]
Online Medical Service Quality	Patients’ evaluation of the design and safety of the online platform and whether the information provided meets the patient’s expectations.	3	1. The online doctor was very quick to reply to me. 2. The online doctor is highly qualified. 3. Online healthcare platform is easy to operate.	[59]
